# The involvement of survival signaling pathways in rubella-virus induced apoptosis

**DOI:** 10.1186/1743-422X-2-1

**Published:** 2005-01-04

**Authors:** Samantha Cooray, Li Jin, Jennifer M Best

**Affiliations:** 1 Enteric, Neurological, and Respiratory Virus Laboratory, Health Protection Agency, 61 Colindale Avenue, London NW9 5HT, UK; 2 Department of Infectious Diseases, Virology Section, Guy's, King's and St. Thomas' School of Medicine, St. Thomas' Hospital, London SE1 7EH, UK; 3 Present address: Department of Virology, 3^rd ^Floor, Wright Flemming Institute, Imperial College Faculty of Medicine, Norfolk Place, London W2 1PG, UK

## Abstract

Rubella virus (RV) causes severe congenital defects when acquired during the first trimester of pregnancy. RV cytopathic effect has been shown to be due to caspase-dependent apoptosis in a number of susceptible cell lines, and it has been suggested that this apoptotic induction could be a causal factor in the development of such defects. Often the outcome of apoptotic stimuli is dependent on apoptotic, proliferative and survival signaling mechanisms in the cell. Therefore we investigated the role of phosphoinositide 3-kinase (PI3K)-Akt survival signaling and Ras-Raf-MEK-ERK proliferative signaling during RV-induced apoptosis in RK13 cells. Increasing levels of phosphorylated ERK, Akt and GSK3β were detected from 24–96 hours post-infection, concomitant with RV-induced apoptotic signals. Inhibition of PI3K-Akt signaling reduced cell viability, and increased the speed and magnitude of RV-induced apoptosis, suggesting that this pathway contributes to cell survival during RV infection. In contrast, inhibition of the Ras-Raf-MEK-ERK pathway impaired RV replication and growth and reduced RV-induced apoptosis, suggesting that the normal cellular growth is required for efficient virus production.

## Introduction

Rubella virus (RV) is the sole member of the *Rubivirus *genus of the *Togaviridae*. It has a positive-sense single stranded RNA genome that is 9762 nucleotides (nt) in length and contains two non-overlapping open-reading frames (ORFs). The 5' proximal ORF encodes the p200 polyprotein precursor for the nonstructural proteins (NSPs) p150 and p90 [1,2]. The 3' proximal ORF encodes the structural proteins: capsid (C), and glycoproteins E1 and E2 [3,4].

RV infection usually causes mild disease with few complications. However, infection during the first trimester of pregnancy results in fetal infection, and in more than 75% of cases this leads to the development of congenital abnormalities. These abnormalities include sensorineural deafness, mental retardation, and congenital heart defects, and are collectively termed congenital rubella syndrome (CRS) [5]. The cellular mechanisms activated by RV, which lead to the disruption of organogenesis, are not fully understood. However, in permissive cell cultures, the cytopathic effect (CPE) of RV has been shown to be due to caspase-dependent apoptosis [6-12]. Apoptosis is a key component of developmental processes in mammals, which functions to delete vestigial structures, control cell number and remodel tissues and organs [13]. Thus, it has been proposed that RV-induced apoptosis may cause irreparable damage to fetal tissues, resulting in the abnormalities observed in CRS [12]. However, the outcome of RV infection is likely to depend on multiple signaling events that control the balance between cell death and cell survival.

Eukaryotic cells contain a large number of mitogen activated protein kinase (MAPK) signaling cascades that are activated in response to growth factors, cytokines and stress stimuli such as viral infection and UV irradiation. In common with apoptotic proteins, MAPKs are highly conserved and ubiquitously expressed [14,15]. These cascades integrate external stimuli and transmit signals to the nucleus resulting in the activation of transcription factors, which regulate expression of genes required for proliferation, differentiation, survival and apoptosis. Two well-studied mitogenic pathways are the phosphoinositide 3-kinase (PI3K)-Akt pathway and the Ras-Raf-MEK-ERK pathway, which are central to cell survival and proliferative signals respectively.

PI3Ks phosphorylate plasma membrane inositol lipids at the 3' position of the inositol ring. These 3'phosphoinsoitides recruit proteins such as Akt and phosphoinositide dependent kinases 1 and 2 (PDK1/2) to the plasma membrane via their pleckstrin homology (PH) domains [16,17]. At the plasma membrane PDK1/2 activate Akt through phosphorylation at Ser^473 ^and Thr^308^. Activated Akt promotes cell survival by phosphorylating and inhibiting a number of pro-apoptotic proteins including BAD, caspase-9, GSK-3β and Forkhead transcription factors [18,19].

The Ras-Raf-MEK-ERK is a classical MAPK pathway where growth factor-receptor interactions trigger intracellular activation of the small G-protein Ras. Ras recruits and directly activates the MAPK kinase kinase (MAPKK) Raf, which phosphorylates and activates the MAPK kinase (MAPKK) MEK1/2, which in turn activate the MAPK ERK1/2. Activated ERK1/2 translocates to the nucleus where it can activate a number of transcription factors including c-*myc*, c-*jun*, and *Elk-1*, which regulate cell cycle progression responses [20].

Activation of PI3K-Akt and Ras-Raf-MEK-ERK signaling cascades during virus infection is thought to play an important role not only in cellular growth and survival, but also in virus replication and growth during both acute and chronic virus infections [21-25]. This study was carried out to examine the role of PI3K-Akt and Ras-Raf-MEK-ERK signaling during RV infection in RK13 cells. The PI3K inhibitor LY294002 and the MEK inhibitor U0126 were used to investigate PI3K-Akt and Ras-Raf-MEK-ERK signaling respectively during RV replication, growth and induction of apoptosis. Apoptosis was measured in RV-infected cells by caspase activity and cell viability assays, DNA fragmentation analysis, and trypan blue exclusion staining. Involvement of PI3K-Akt and Raf-Raf-MEK-ERK signaling in RV-induced apoptosis was also examined by expression of constitutively active Akt and MEK in RV-infected cells.

## Results

### Phosphorylation of Akt, ERK1/2 and their downstream targets during RV infection

**Figure 1:**
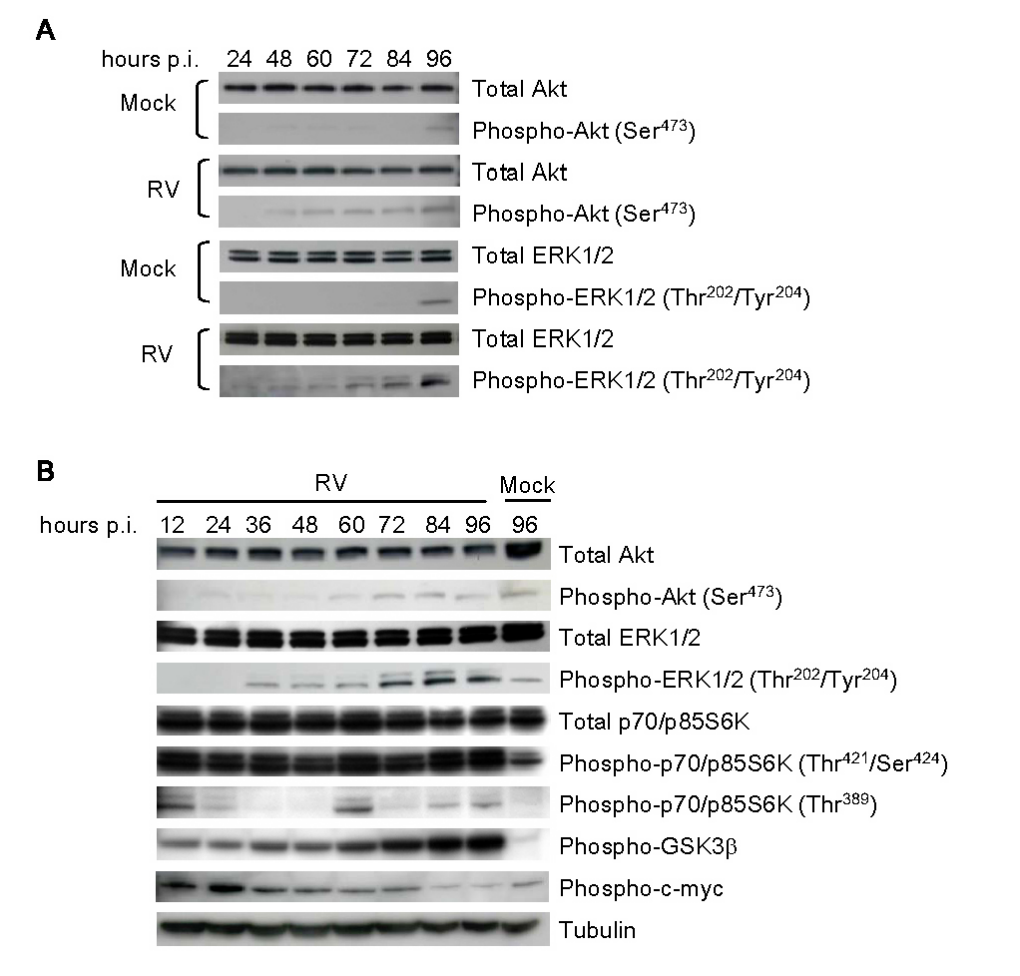
Kinase phosphorylation during RV infection. Serum-starved RK13 cells were mock infected or infected with RV at an m.o.i. of 4 PFU/cell. At indicated time points cell lysates were collected and proteins (30 μg/lane) were separated by SDS-PAGE, and analysed by Western blotting using phospho-specific antibodies. Blots were also probed with anti-tubulin antibody to demonstrate equal loading. A – Total and phosphorylated Akt and ERK (24–96 hours p.i.). B – Total and phosphorylated Akt, ERK, and p70S6K, and phosphorylated GSK-3β and c-myc. The data were consistently repeated in two independent experiments.

The effect of RV infection on PI3K-Akt and Ras-Raf-MEK-ERK pathways was investigated by examining the expression and phosphorylation profiles of Akt, ERK1/2 and their downstream targets. Cell lysates from RV and mock infected RK13 cells were collected 12–96 hours post-infection (p.i.), separated by SDS-PAGE, and analyzed for total and phosphorylated Akt and ERK1/2 by Western blotting. Phosphorylated Akt and ERK1/2 could be detected in RV-infected cells from 48 hours p.i., and band intensity increased from 48–96 hours p.i. compared to total levels (Fig. 1A). Phosphorylated Akt and ERK2 (but not ERK1) were detected in the mock-infected cells at 96 hours p.i. but not before, whereas total levels of Akt and ERK 1/2 were detectable at all time points (Fig. 1A). Treatment of RV-infected cells with PI3K inhibitor LY294002 and MEK1/2 inhibitor U0126 completely inhibited activation of Akt and ERK1/2 respectively (data not shown).

The phosphorylation of Akt and ERK and their downstream targets p70S6K, GSK-3β, c-*myc *and BAD were also examined by Western blotting between 12–96 hours p.i. (Fig. 1B). Phosphorylated Akt and ERK1/2 were detectable in RV-infected cells at 48 and 36 hours p.i. respectively. p70S6K is phosphorylated by FRAP/mTOR downstream of Akt at Thr^389 ^and at Thr^421^/Ser^42^, downstream of the Ras-Raf-MEK-ERK pathway. Phosphorylation at Thr^389 ^was observed at 12, 24, 60, 84 and 96 hours p.i. (Fig. 1B). Phosphorylation of the Thr^421^/Ser^42 ^site was observed at all time points, although increases in band intensity could be seen at 12, 24, 60, 84 and 96 hours p.i., mirroring the phosphorylation at Thr^389^. Phosphorylation of Thr^421^/Ser^424 ^but not Thr^389 ^was observed in the mock-infected cells, albeit at a lower level than in RV-infected cells.

The phosphorylation of GSK-3β, downstream of Akt, increased from 12 and 96 hours p.i. and was similar to that of Akt. Phosphorylation of BAD, another substrate for Akt, however, could not be detected in RV-infected or mock-infected cells. The phosphorylation of c-*myc*, a transcription factor activated by ERK1/2 phosphorylation, decreased between 12 and 96 hours p.i., in contrast to the phosphorylation profile of ERK1/2. GSK-3β and c-*myc *were also detectable in the mock-infected cells at 96 hours p.i.

### The effects of LY294002 and U0126 on cell viability in RV-infected cells

RV induces apoptosis in RK13 cells with characteristic morphological and biochemical features [6,8,9]. The XTT assay was used to examine the effect of RV infection and LY29002 and U0126 treatment on cellular metabolism over time. XTT is a tetrazolium salt, which is cleaved by the succinate dehydrogenase system of mitochondria in metabolically active cells, to yield a soluble orange formazan product. A decrease in the intensity of formazan was used to monitor changes in cellular metabolism and cell viability in RV-infected cells by spectroscopy.

**Figure 2:**
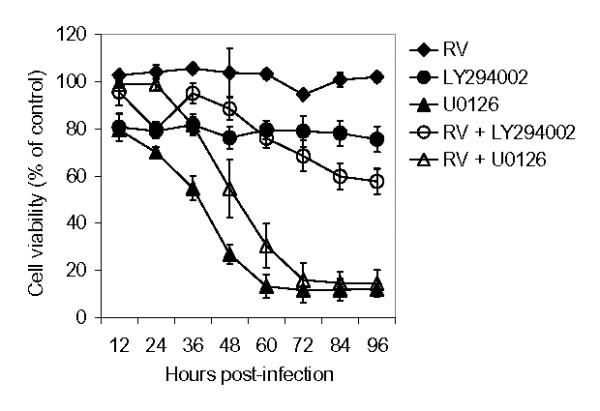
The effect of PI3K and MEK1/2 inhibition on cell viability during RV infection. Serum-starved RK13 cells were mock infected or infected with RV at an m.o.i of 4 PFU/cell with or without LY294002 (30 μM) or U0126 (15 μM). At indicated time points cell viability was determined by XTT assay. Tetrazolium salt (XTT) and electron coupling reagent were added directly to cells, and after 24 hours the absorbance at 405–690 nm was determined. Data represent mean ± S.E. from three independent experiments.

Cellular viability during RV infection did not appear to be disrupted, supporting previous observations which reported that a large number of monolayer cells remain in tact and do not rapidly undergo apoptosis in RV infected cells [9,12] (Fig. 2). LY294002 treatment of RK13 cells reduced cell viability by 20%, which remained constant throughout the 12–96 hour period. Cell viability was reduced to 60% in the presence of both RV and LY294002. Thus the combined effect of PI3K inhibition and RV-infection caused a significant reduction in cell viability.

As Ras-Raf-MEK-ERK signaling is crucial to the regulation of cell growth in many cell lines, inhibition of this pathway often has detrimental effects. A typical dose-response curve can be seen with MEK inhibitor U0126 in RK13 cells, with cell viability completely abolished by 60–72 hours p.i. (Fig. 2). With the addition of RV, the U0126 curve moved to the right, the effect of the drug was delayed by approximately 12 hours.

### Inhibition of PI3K results in an increase in the speed and magnitude of RV-induced apoptosis

**Figure 3:**
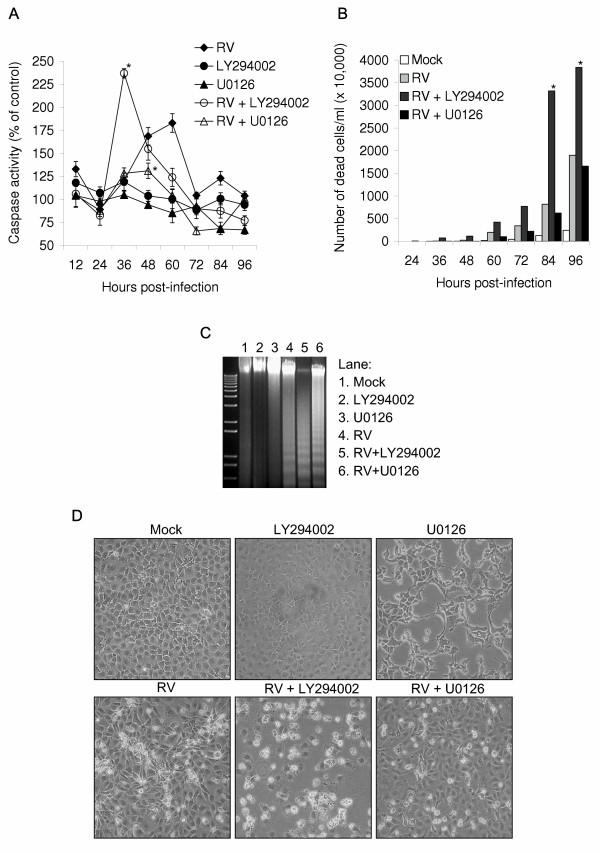
The effect of PI3K and MEK1/2 inhibition on RV-induced apoptosis. Serum-starved RK13 cells were mock infected or infected with RV at an m.o.i of 4 PFU/cell with or without LY294002 (30 μM) or U0126 (15 μM). Cells were harvested and analyzed for markers of apoptosis. A – At indicated time points, cell lysates were collected and incubated with artificial caspase substrate Ac-DEVD-pNA. Free pNA due to caspase cleavage was measured at an absorbance of 405 nm. Data represent mean ± S.E. from three experiments, *P < 0.05. B – The number of measurable dead floating cells in the cell culture supernatant was determined by trypan blue exclusion staining at indicated time points. Data represent mean ± S.E. from three experiments, *P < 0.05. C – Total DNA was extracted from detached and monolayer cells at 72 hours p.i. and apoptotic DNA fragments were resolved on a 1.5% agarose gel, stained with ethidium bromide, and visualized using UV transillumination. Molecular size markers were run in the *left hand lane*. D – Light microscopy photographs of cell monolayers at 72 hours p.i., at a magnification of 20X.

To evaluate the role of PI3K-dependent signaling during RV infection, the effects of PI3K inhibitor LY294002 on the development of RV-induced apoptosis were examined, 12–96 hours p.i., by caspase activity assay, trypan blue exclusion staining, DNA fragmentation and light microscopy. (Fig. 3A–D). RV-induced apoptotic signaling has been reported to occur between 12–24 hours p.i., with peak caspase activity occurring around 72 hours p.i. at a multiplicity of infection (MOI) of 3 PFU/cell [6]. Fig. 3A shows that with a MOI of 4 PFU/cell the peak of RV-induced caspase activity occurs earlier at 60 hours p.i. When RV infection was carried out in the presence of LY294002, the maximum caspase activity increased by 53.9 % (P < 0.05) and occurred 12 hours earlier than with RV alone (Fig. 3A).

This increase in speed and magnitude of RV-induced apoptosis is more strikingly observed in Fig. 3B, which shows the number of dead floating cells by trypan exclusion staining in the culture supernatant fluid of RV infected and LY294002 treated cells. LY294002 treatment doubles (and at 84 hours p.i. triples) the number of floating cells produced in RV-infected cells. Increases in the number of apoptotic floating cells are statistically significant at 84 and 96 hours p.i. (P < 0.05). Fragmented DNA patterns can be seen at 72 hours p.i. with both RV and RV in the presence of LY294002 (Fig. 3C). However, the interesting feature of these apoptotic ladders is that in RV-infected cells, a significant proportion of genomic DNA is still intact, whereas when RV-infected cells are also exposed to LY294002, the majority of the genomic DNA is fragmented. The morphological changes caused by RV-infection and LY294002 were examined by light microscopy (Fig. 3D). At 72 hours p.i. CPE and induction of apoptosis by RV can be clearly seen. RV-induced CPE is characterized in the earlier stages by clumps of apoptotic cells, surrounded by healthy cells. In the later stages the cell sheet is completely destroyed and the majority of cells have become apoptotic floaters [6]. In the presence of LY294002, RV-infected cells are almost all dead by 72 hours p.i., resembling the later stages of RV-induced CPE.

LY294002-only treatment of RK13 cells did not induce apoptosis as evidenced by the lack of caspase activity (Fig. 3A), DNA fragmentation (Fig. 3C), and measurable floating cells (data not shown). Morphological examination of LY294002 treated RK13 cells show the cell monolayers were in tact with no visible cytotoxicity (Fig. 3D).

### Inhibition of MEK1/2 reduces RV-induced apoptosis

The role of Ras-Raf-MEK-ERK signaling in RV-induced apoptosis was investigated using MEK inhibitor U0126 as described above for LY294002 (Fig. 3A–D). U0126 treatment reduced caspase activity in RV-infected cells by 51.9% (P < 0.05), with a low peak occurring at 48 hours p.i. (Fig. 3A). The number of dead floating cells in RV and U0126-treated cells was slightly lower than in RV-infected cells at all time points (Fig. 3B). DNA fragmentation was observed in both RV-infected cells and RV in the presence of U0126 (Fig. 3C), although the presence of the drug also appeared to result in smearing of high molecular weight DNA, characteristic of necrosis [26,27]. The detrimental effect of U0126 on RK13 cell morphology is shown in Fig. 3D; this correlates with the rapid decline in cell viability.

### Inhibition of MEK1/2 inhibits RV replication and growth

**Figure 4:**
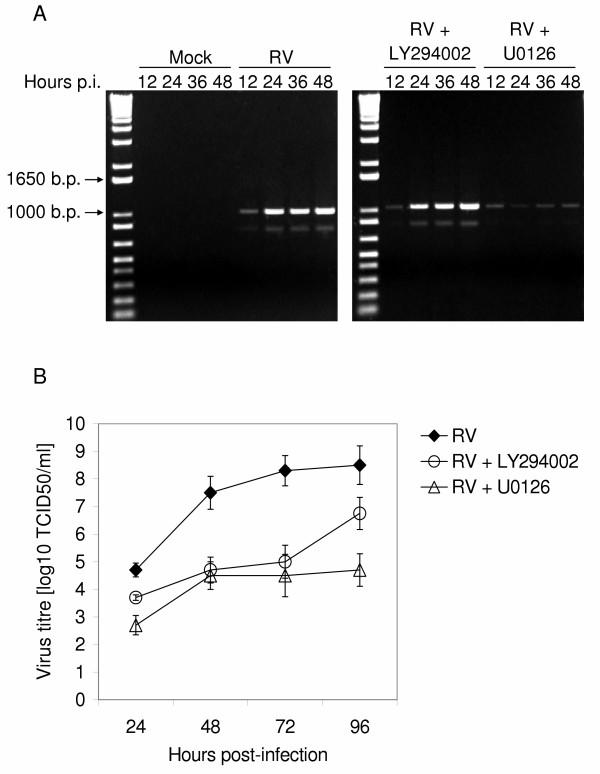
The effect of PI3K and MEK1/2 inhibition on RV growth and replication. Serum-starved RK13 cells were infected with RV at an m.o.i of 4 PFU/cell with or without LY294002 (30 μM) or U0126 (15 μM). Cell culture supernatants were extracted from cells at indicated time points. A – RV RNA was extracted from virus-infected cell culture supernatants and the capsid gene was amplified by RT-PCR as described under 'Experimental Procedures'. B – Monolayers of RK13 cells in 96-well plates were infected with RV-infected cell culture supernatants, and virus titers were determined using the TCID_50 _assay. Results are representative of a least two independent experiments.

To examine the effect of LY294002 and U0126 on RV replication and growth, RV-infected and drug-treated cell culture supernatants were tested for RV capsid gene expression by RT-PCR, and virus growth by TCID_50 _assay 24–96 hours p.i.. The capsid gene is the first gene to be transcribed from the second ORF encoding the structural proteins. Therefore detection of capsid RNA by RT-PCR is a good measure of RV replication [1,28]. In RV-infected cells simultaneously treated with LY294002, levels of RV capsid RNA increased over time, as in RV-infected cells (Fig. 4A). In the presence of U0126, however, levels of capsid RNA were reduced, and remained lower than that seen at 24 hours p.i. in RV-infected cells.

Both LY294002 and U0126 affected virus growth (Fig. 4B). During RV-infection of RK13 cells with 4 PFU/cell of virus, virus titers reached 10^8 ^TCID_50_/ml by 96 hours p.i. However, in the presence of U0126 the titer was approximately 10^2 ^lower at 24 hours p.i., 10^3 ^lower at 48 hours p.i., and 10^4 ^lower at 72–96 hours p.i. LY294002 reduced virus growth to a similar extent, but unlike with U0126, by 96 hours p.i. the virus titer recovered slightly.

### Constitutively active Akt and MEK1/2 enhance RV-induced apoptosis

**Figure 5:**
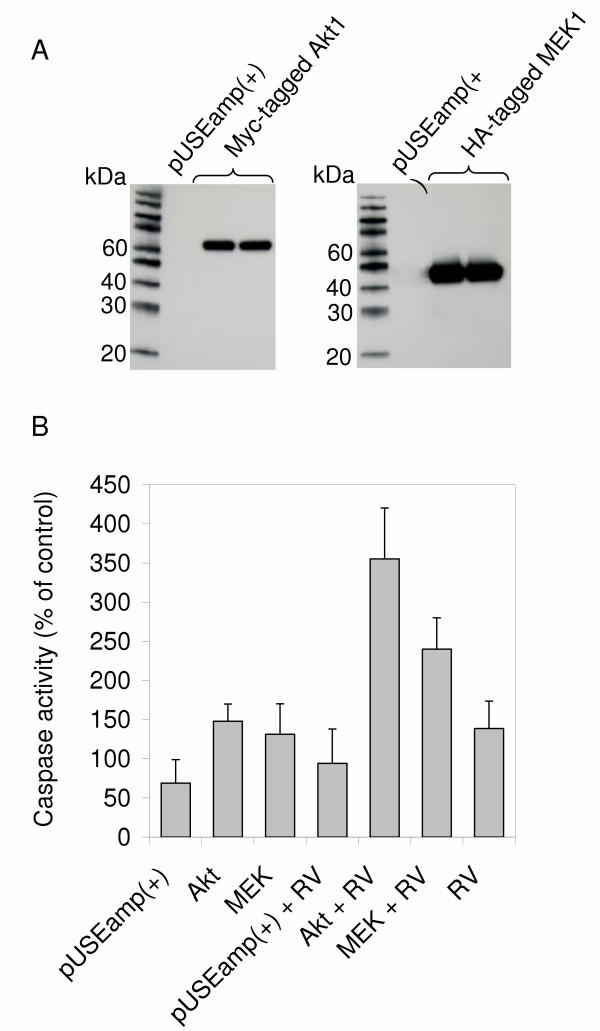
Over-expression of Akt and MEK enhances RV-induced apoptosis. RK13 cells were transfected with eukaryotic expression vector pUSEamp(+) containing constitutively active HA-tagged MEK1 or myristoylated myc-tagged Akt1 under the control of a CMV promoter, or with an empty pUSEamp(+) control. A – Expression of MEK1 and Akt1 was determined by Western blotting. Cell lysates were collected 24 hours post-transfection and 30 μg protein separated by SDS-PAGE and transferred to nitrocellulose membranes. MEK1 and Akt1 were detected by anti-HA and anti-myc antibodies respectively. B – RK13 cells in 6-well plates were transfected with Akt, MEK or pUSEamp(+) control constructs for 24 hours and subsequently infected with RV or mock-infected. 24 hours later cell lysates were collected and tested for caspase activity using artificial caspase substrate Ac-DEVD-pNA.

To determine the importance of PI3K-Akt and Ras-Raf-MEK-ERK in the transduction of cell survival and proliferative mechanisms during RV-infection, RK13 cells were transiently transfected with constitutively active forms Akt and MEK. Significant expression of both proteins was seen after 24 hours (Fig. 5A). Over-expression of both activated Akt and MEK enhanced RV-induced caspase activity (Fig. 5B). RV infection in the presence of the empty pUSEamp(+) control vector slightly decreased caspase activity. Caspase activity following Lipofectamine treatment alone or pUSEamp(+) transfection was below that of the mock-infected cells (data not shown).

## Discussion

We have previously shown that RV induces caspase activation during the early stages of infection *in vitro*, prior to the appearance of morphological apoptotic changes [6]. In this study we demonstrated that, in common with other viruses such as Coxsackievirus B3 virus, human cytomegalovirus, influenza virus A, and respiratory syncitial virus (RSV) (Cooray, 2004; Johnson et al., 2001; Opavsky et al., 2001; Pleschka et al., 2001), signaling downstream of PI3K stimulates a survival response in the cell following RV infection and that signaling downstream of MEK1/2 is required for RV replication, growth and induction of apoptosis.

Analysis of phosphorylation profiles during RV infection demonstrated that the presence of the virus stimulated an increase in the phosphorylation of ERK1/2, Akt, and Akt target GSK-3β over time. The presence of phosphorylated Akt (and occasionally ERK2) at 96 hours p.i. in the mock-infected cells, suggests that cell survival mechanisms may be activated in older uninfected cell cultures. The phosphorylation pattern of downstream target p70S6K did not follow that of Akt and ERK1/2. Apart from being phosphorylated by ERK1/2 and mTOR/FRAP downstream of Akt, p70S6K can be phosphorylated by an array of different proline-directed kinases, including PDK1, PKCζ, JNK and cdc2 which may explain this difference [29-33].

The phosphorylation of c-*myc*, a downstream target of ERK1/2, did not follow the same pattern. Levels of phosphorylated c-*myc *decreased as infection progressed, which was probably due to its targeted degradation or the action of cellular phosphatases. RV infection has been observed to slow cell cycle progression both *in vivo *and *in vitro *[12,34]. As c-*myc *is a transcription factor that stimulates cell cycle progression, its de-phosphorylation or degradation as RV infection progresses supports these observations. The expression and activity of c-*myc *and other downstream transcription factors in relation to the cell cycle during RV-infection requires further investigation. Phosphorylation of BAD, downstream of Akt, could not be detected in RV-infected cells (data not shown). However, BAD is not ubiquitously expressed and therefore may not be produced in the rabbit kidney epithelial cells (RK13) used [16].

Inhibition of PI3K signaling with LY294006 significantly increased the speed and magnitude of RV-induced apoptosis as shown by increased caspase activity, dead floating cells, apoptotic laddering of genomic DNA and decreased cell viability. Thus, RV-induced apoptotic signaling appears to be held in check by host cell survival signals downstream of PI3K. Although inhibition of PI3K did not affect RV replication, virus growth was affected. The speed of apoptotic monolayer death may have prevented production of optimal virus titers.

The importance of PI3K survival signaling has been observed with other viruses. Recently phosphorylation of Akt, GKS3β and PKCζ (another downstream target of PI3K signaling), has been demonstrated in Vero E6 cells early during infection with severe acute respiratory syndrome (SARS)-associated corona virus (CoV) [35]. However, unlike in this study the survival response due to PI3K-Akt signaling was deemed to be weak, as LY294002 treatment did not result in an increase in apoptotic DNA laddering. PI3K, Akt and NFκB have also been shown to be activated prior to epithelial cell apoptosis in RSV-infected cells [36]. As with RV, inhibition of PI3K increased the speed and magnitude of RSV-induced apoptosis, although host-cell survival was suggested to occur prior to apoptotic signaling, as opposed to RV where caspase activation and Akt phosphorylation occur concomitantly [6]. PI3K-Akt signaling has also been shown to reduce coxsackievirus B3 (CVB3)-induced apoptosis. However, in contrast to RSV, the replication of CVB3 was also reduced, suggesting that PI3K-Akt survival signals may also serve as a defense mechanism against virus infection [37].

Inhibition of the MEK1/2 in RK13 cells by U0126 resulted in necrotic monolayer destruction and a significant decrease in cell viability. XTT assay and light microscopy demonstrated that RV infection appeared to delay the effect of U0126. As discussed above, RV infection stimulates ERK activity, downstream of MEK, and may therefore counteract the effect of the inhibitor. Despite this, U0126 impaired RV replication, growth, and induction of apoptosis. Therefore it appears that although RV infection slows the cell cycle progression, cells must be cycling and metabolizing normally for RV replication to occur.

ERK1/2 phosphorylation has also been observed during infection with a number of other viruses, and inhibition of ERK1/2 signaling by U0126 has consistently been shown to be detrimental to virus growth. Infection of Jurkat cells with CVB3, for example, leads to up-regulation of ERK1/2 phosphorylation, and elevated levels of phosphorylated ERK1/2 have been observed in the myocardium of mice susceptible to CVB3-induced myocarditis [38]. Treatment of cultured cells with U0126 reduced CVB3 titers and inhibited the release of virus progeny [38,39]. Similarly, HCMV infection in human embryonic lung fibroblasts (HELs) has been shown to stimulate biphasic activation of MEK1/2 and ERK1/2, and treatment of infected cells with U0126 reduced viral DNA replication, protein production and virus titer [40]. Influenza A virus infection *in vitro *has also been shown to stimulate biphasic activation of MEK1/2 and ERK1/2, and U0126 treatment prevented export of ribonucleoprotein complexes from the nucleus and inhibited virus production [24]. Inhibition of MEK1/2 during HIV infection has been demonstrated to reduce infectivity, but unlike the other viruses mentioned herein, did not affect protein levels or virus production [25]. These findings, along with the results of this study, suggest that signaling downstream of MEK1/2 and ERK1/2 is important for viral infectivity and efficient virus replication and growth *in vitro*.

Over-expression of Akt and MEK1/2 increased RV-induced caspase activity in RK13 cells. This response was not due to the transfection procedure, as the increase in caspase activity was not observed in the pUSEamp(+) or lipofectamine controls. Such a response is also seen in malignant cells, which are more readily killed by apoptotic stimuli. Thus, the over-expression of these mitogenic pathways may have resulted in a cell survival response whereby a negative feedback loop occurred that sensitized cells to RV-induced apoptosis. In order to study this further, it would be necessary to construct stable cell lines over-expressing active Akt and ERK1/2 as well as their dominant negative mutants and other signaling proteins.

It is clear from the results of this and previous studies that the outcome of RV infection *in vitro *depends on numerous signaling events. It has been suggested that RV capsid protein, when anchored to the ER can independently induce apoptosis in culture (Duncan et. al, 2000). However this has not been confirmed by other groups and there is conflicting evidence that virus replication and the presence of the RV NSPs (which are necessary for replication) is required [10,12,41]. Interestingly the NSP p90 has been shown to interact with the retinoblastoma (pRB) cell cycle-regulatory protein and the cytokinesis regulatory protein citron-K kinase (CK), and it has been suggested that this may perturb the cell cycle [42,43]. How these interactions interfere with signaling pathways and modulate cellular responses, however, remains to be determined.

In relation to CRS, study of the expression and localization of apoptotic and mitogen activated signaling proteins in RV-infected fetal tissues would be necessary to confirm the theory that the pathogenesis of the disease is related to perturbation of the cell cycle. However as CRS is now rare in the UK and work with fetal tissues is tightly regulated, such a study would be hard to carry out. *In vivo *studies are difficult, as a reliable animal model does not exist for CRS. However, it may be possible to extrapolate findings from cell culture systems. We used RK13 cells because they are the best cells in which to detect rubella-induced apoptosis; further studies are required to confirm our findings in primary human embryonic cells.

## Materials and methods

### Chemical Compounds

Stock concentrations of PI3K inhibitor LY294002 [2-(4-Morpholinyl)-8-phenyl-1-4H-1-benzopyran-4-one] and MAPK/MEK inhibitor U0126 [1, 4-Diamino-2, 3-dicyano-1, 4-bis (2-aminophenylthio) butadiene] (Calbiochem, UK) were made up in dimethyl sulfoxide (DMSO). In all experiments LY294002 and U0126 were used at concentrations of 30 μM and 15 μM respectively.

### Cell Culture & Viral Infection

Mycoplasma-free rabbit kidney epithelial (RK13) cells were obtained from the European Collection of Cell Cultures and cultured as previously described (3). RV (wild type strain RN) was propagated as previously described (3). For infection, cells were grown to confluence in minimal essential medium (MEM) supplemented with 15 mM L-glutamine and 5% FCS (v/v) (Invitrogen, UK) at 37°C in 5% CO_2 _in air, and serum starved overnight. Cells were infected with RV at a MOI of 4 plaque forming units (PFU) per cell and maintained in MEM with 1% FCS until harvested at indicated time points. Where appropriate kinase inhibitors (LY294002 and U0126) were added to the media at the same time as the virus, and were present during subsequent incubation periods. Mock-infected cells were treated and harvested in the same manner as those infected, except that MEM without virus was used during the infection. RV titers, in the presence of inhibitors, were determined by TCID_50 _assay in RK13 cells as the sample number was too large to perform plaque assays. Inhibitor, virus and serum concentrations were optimized to ensure that the effect of both the virus and the inhibitors could be monitored.

### Transfection

Control and expression plasmids [pUSEamp(+), and constitutively active HA-Akt1 and Myc-MEK1 in pUSEamp(+)] were purchased from Upstate Biotechnology Inc. (UK). RK13 cells were grown to confluence in 25 cm^2 ^tissue culture flasks and transiently transfected with 0.25 μg of control or expression plasmids. Tranfections were carried out in serum-free MEM using Lipofectamine Plus (Invitrogen, UK), according to the manufacturer's instructions. For optimal transfection, cell monolayers were incubated with the DNA-liposome mixture for 5 hours at 37°C. Following transfection, the DNA liposome complexes were removed and replaced with fresh medium. After 24 hours, RV was added to cells, which were maintained on MEM with 1% serum (as above). After an additional 24 hours, cells were analyzed for protein expression by Western blot analysis, and for apoptosis by caspase activity assay.

### Western Blot Analysis

Polyclonal anti-PI3K p85, anti-HA Tag, anti-myc Tag, and monoclonal anti-β-tubulin antibodies were from Upstate Biotechnology inc. (UK). Polyclonal anti-caspase-3 antibody was from Sigma (UK). All other primary antibodies were purchased from Cell Signaling Technology (UK). Cells were treated as described above and at indicated times post-infection (p.i.), washed in PBS and harvested in cell lysis buffer [50 mM Tris, 150 mM NaCl, 1% Triton-X-100, 2 mM EDTA, 2 mM EGTA, 100 μM protease inhibitor cocktail, and 100 μM each of phosphatase inhibitor cocktails 1 and 2 (Sigma, UK)]. Protein concentrations were determined using the BioRad assay (BioRad, Hemel Hemstead, UK), and equal protein loading was determined by Coomassie staining (Invitrogen, Paisley, Scotland). Lysates were electrophoresed on 12% Bis-Tris polyacrylamide gels (Invitrogen, UK) and transferred onto Hybond™ ECL nitrocellulose or PVDF membranes (Amersham Biosciences, UK). Membranes were blocked with 5% non-fat dried milk in PBS containing 0.1% Tween-20, and subsequently incubated with primary antibody (1:1000) overnight at 4°C. Specific antibody binding was detected using horseradish peroxidase conjugated anti-rabbit or anti-mouse IgG (1:2000) (Dako, UK), and immunoreactive bands were visualized using the ECL detection system according the manufacturer's instructions (Amersham Biosciences, UK).

### XTT Assay

RK13 cells were grown to confluence in 96-well tissue culture plates at 37°C in 5% CO_2 _in air. Cells were treated, in a final volume of 100 μl, with RV and kinase inhibitors as described above. At indicated times p.i., 50 μl of labeling mixture containing XTT (sodium 3'- [1-(phenylaminocarbonyl)-3, 4-tetrazolium]-bis (4-methoxy-6-nitro) and coupling reagent PMS (N-methyl dibenzopyrazine methyl sulphate) (Roche Applied Science, Mannheim, Germany) was added directly to the wells to give final concentrations of 0.3 mg/ml and 2.5 μg/ml respectively. Plates were incubated in a humidified atmosphere (37°C, 5% CO_2_) for 24 hours. The absorbance of the formazan product was measured at a test wavelength of 450 nm and a reference wavelength of 690 nm.

### Caspase Activity Assay

DEVD specific caspase activity assay (Promega, UK) was carried out as previously described (3). Briefly, RK13 cells were grown to confluence, and treated with RV, LY294002, and U0126 (as above). Cell lysates were collected at indicated times p.i. and stored at -70°C until required. For the assay, lysates were incubated with colorimetric substrate DEVD-p-NA for 4 hours at 37°C, and absorbance of free pNA cleaved by endogenous caspases-3 and -7 was measured at 405 nm.

### DNA Fragmentation Analysis

Analysis of apoptotic DNA fragmentation was carried out as previously described (3). Briefly, RK13 cells in 6-well plates were treated with RV, LY294002 and U0126 as above, and harvested 72 hours p.i. Total cellular DNA was extracted from 2 × 10^6 ^cells according to the manufacturer's instructions (Calbiochem, Nottingham, UK). Nucleic acids were precipitated using 3 M sodium acetate, 2-propanol, and ethanol. DNA pellets were dried and re-suspended in 10 mM Tris pH 7.5, 1 mM EDTA. Ladder fragments were electrophoretically separated on 1.5% Tris-Acetate EDTA (TAE) agarose gels. Gels were stained in ethidium bromide solution (5 mg/ml) and fragmented DNA was visualized under UV light.

### Examination of floating cells

Floating dead cells in the supernatant following infection with RV or drug treatment (as described above) were quantified by trypan blue exclusion staining. The morphological changes to the cells were examined by light microscopy using a Nikon Eclipse TS100 light microscope. Pictures of cells were taken at a magnification of 20X using a Nikon COOLPIX 4500 digital camera and processed with Adobe Photoshop 7.0 software.

### RV Capsid RT-PCR

Total RNA was extracted from 100 μl tissue culture supernatants, collected at indicated times p.i., using a silica-guanidinium isothiocyanate method [44]. Prior to reverse transcription, RV RNA was heated to 95°C for 1 minute and kept on ice. RNA was transcribed to cDNA using Superscript III RNase H^- ^reverse transcriptase (Invitrogen, UK). Reverse transcription was performed in 20 μl reaction volumes containing 200 U enzyme, 10 μl sample RNA, 0.5 mM of each dNTP, and 5 pmoles external reverse primer (5'-CCTGTACGTGGGGCCTTTAA-3'). RNA bound to cDNA in RNA-DNA hybrids was removed by incubation of the cDNA with RNase H (Roche Diagnostics, UK) for 20 minutes at 37°C. PCR amplification was carried out using a GC-Rich PCR System (Roche Diagnostics, UK). In the PCR reaction 10 μl cDNA was added to 40 μl of PCR reaction mix to give final concentrations of 1X GC-Rich PCR buffer, 1.5 mM MgCl_2_, 0.2 mM each dNTP, 0.5 M GC-rich resolution solution™, 0.5 pmole of forward and reverse primers (5'-TAGGAGGTGCCGCCATATCA-3' and 5'-CCTGTACGTGGGGCCTTTAA-3' respectively), and 2U Taq polymerase and a mixture of proof-reading polymerases. The cycling conditions, as recommended by the manufacturer were: 95°C for 3 minutes followed by 10 cycles of 95°C for 30s, 57°C for 30s, 72°C for 1 minute; and 25 cycles of 95°C for 30s, 57°C for 30s, 72°C for 1 minute (plus an additional 5 seconds per cycle), and a final extension of 72°C for 7 minutes. Amplified capsid product (1053 b.p.) was electrophoretically separated on 1% Tris-Borate (TBE) agarose gels, stained with ethidium bromide solution (5 mg/ml) and visualized under UV light.

## Authors' Contributions

SC conceived of the study, carried out the virological and biochemical assays and drafted the manuscript. JL participated in the design of the study. JMB participated in design and coordination of the study and helped to draft the manuscript. All authors read and approved the final manuscript.
